# Supplementary material for: Complexome profiling of the Chlamydomonas psb28 mutant reveals TEF5 as an early PSII assembly factor
Source: Plant Cell. 2025 Mar 17;37(6):koaf055. doi: 10.1093/plcell/koaf055 (PMC12164590; doi:10.1093/plcell/koaf055)
Supplement: koaf055_Supplementary_Data [file koaf055_supplementary_data.zip › psb28_interactive.zip!/plots/intensity/qualityPlots/peptides/LHCA6_Cre06.g278213.t1.1.html]

### Cre06.g278213.t1.1

Trivial name: LHCA6  
SeqCoverage [median]: 73.20  
Mass: 27.78 kDa  
  
 

| Sequence | MissedCleavage | Mass [Da] | wtIntensity | muIntensity | Score |
| --- | --- | --- | --- | --- | --- |
| AAVVPGQAVAPPCK | 0 | 1306.71 | 6181.35 | 0.00 | 46.00 |
| ASSRPLWLPGSTPPAHLK | 1 | 1914.05 | 205364.15 | 82726.68 | 70.33 |
| ASSRPLWLPGSTPPAHLKGDLPGDFGFDPLGLGANAESLK | 2 | 4085.10 | 21250.64 | 0.00 | 79.67 |
| ESELVHSR | 0 | 955.47 | 303455.23 | 102556.17 | 248.83 |
| GDLPGDFGFDPLGLGANAESLK | 0 | 2189.06 | 380509.21 | 89275.87 | 202.32 |
| GDLPGDFGFDPLGLGANAESLKWFK | 1 | 2650.31 | 2465.06 | 0.00 | 47.33 |
| GPIAALQEHLADPWGTTIFSK | 0 | 2251.16 | 86135.01 | 20887.98 | 183.60 |
| IPASVSYK | 0 | 863.48 | 647236.21 | 191148.78 | 145.34 |
| KPGSVDQDPIFSQYK | 1 | 1707.85 | 1461097.58 | 855492.54 | 180.70 |
| LAMLAFVGFVMAAQVTGK | 0 | 1852.99 | 139653.55 | 31203.07 | 180.74 |
| LPPHEVGYPGGVFAPFIPGDLAELK | 0 | 2619.37 | 388200.22 | 201669.05 | 95.21 |
| LPPHEVGYPGGVFAPFIPGDLAELKVK | 1 | 2846.54 | 13183.33 | 0.00 | 50.05 |
| NGRLAMLAFVGFVMAAQVTGK | 1 | 2180.16 | 1133.94 | 0.00 | 45.05 |
| PGSVDQDPIFSQYK | 0 | 1579.75 | 2570.75 | 816.98 | 136.38 |
| PLWLPGSTPPAHLK | 0 | 1512.85 | 85755.10 | 19552.72 | 110.38 |
| WAMAAVAGILVQEIVRPDVFWYNAGK | 1 | 2903.52 | 2963.48 | 0.00 | 106.43 |
| WFKESELVHSR | 1 | 1416.71 | 49294.11 | 4645.11 | 120.57 |
| WQDLRKPGSVDQDPIFSQYK | 2 | 2406.20 | 11664.00 | 5860.51 | 80.24 |
|  |  |  | 3808112.9 | 1605835.5 |  |
